# Dynamics of circulating microRNAs as a novel indicator of clinical response to neoadjuvant chemotherapy in breast cancer

**DOI:** 10.1002/cam4.1723

**Published:** 2018-08-11

**Authors:** Wenjie Zhu, Mei Liu, Ying Fan, Fei Ma, Ningzhi Xu, Binghe Xu

**Affiliations:** ^1^ National Cancer Center/National Clinical Research Center for Cancer/Cancer Hospital Chinese Academy of Medical Sciences and Peking Union Medical College Beijing China; ^2^ Laboratory of Cell and Molecular Biology & State Key Laboratory of Molecular Oncology National Cancer Center/National Clinical Research Center for Cancer/Cancer Hospital Chinese Academy of Medical Sciences and Peking Union Medical College Beijing China

**Keywords:** breast cancer, circulating miRNAs, dynamics, neoadjuvant chemotherapy, sensitivity

## Abstract

**Background:**

Circulating microRNAs (miRNAs) have been indicated as predictive biomarkers in breast cancer. We aimed to explore the association of plasma miRNA dynamics with response to neoadjuvant chemotherapy (NCT) and disclose early markers for predicting sensitivity.

**Methods:**

One hundred and nine patients with operable or locally advanced breast cancer, who participated in a prospective clinical trial and received NCT, were analyzed. Blood samples were collected before random assignment, after two cycles of chemotherapy (C2) and before surgery. Based on their clinical response, the patients were defined as chemo‐sensitive or insensitive. First, baseline and preoperative samples of selected cases from both groups were screened via TaqMan miRNA array for candidate miRNAs. Afterward all the biospecimens were tested for the candidate miRNAs (miR‐222, miR‐20a, miR‐451, miR‐9, miR‐34a, miR‐155, and miR‐145) by quantitative real‐time PCR. Finally, logistic regression model was utilized to determine the predictive value of baseline/C2 expression of these miRNAs.

**Results:**

Based on the results of microRNA profiling, seven miRNAs were selected for further validation. In the HR+/HER2‐ cohort (n = 51) dynamics of three miRNAs, including miR‐222, miR‐20a, and miR‐451, were associated with chemo‐sensitivity. Importantly, across all the three subtypes we consistently identified chemo‐induced decrease in plasma miR‐34a in the insensitive patients. Finally, baseline miR‐222 overexpression (OR = 6.422, *P* = 0.049), C2 miR‐20a up‐regulation (OR = 0.144, *P* = 0.021) and C2 miR‐451 down‐regulation (OR = 8.213, *P* = 0.012) were predictive markers of response to NCT in HR+/HER2‐ breast cancer.

**Conclusions:**

We described that dynamics of circulating miRNAs might help predict clinical response to NCT in breast cancer.

## 1 BACKGROUND

Paradigm shifts to neoadjuvant chemotherapy (NCT) for locally advanced breast cancer and in recent years expanded use of NCT has been seen in operable early‐stage cases. Initial trials raised concerns that NCT increased the risk of local recurrence which was probably due to subsequent management with radiotherapy instead of surgery in some series.^1^, ^2^, ^3^, ^4^ Later studies established that responders who achieve pathological complete remission (pCR) after NCT have significantly improved outcomes compared with nonresponders,^5^, ^6^ which provides rationale for identification of predictive biomarkers. Use of such biomarkers can reduce chemotherapy‐related toxicities and potential risk of distant metastasis and facilitate tailored clinical management. Despite global efforts to discover predictive biomarkers for NCT in breast cancer, till now there has been no clinically validated method for reliable prediction of chemotherapeutic (non)responders.^7^, ^8^, ^9^

MicroRNAs (miRNAs) are short (18‐25 nucleic acids) noncoding single‐strand RNAs that can bind to the 3′‐untranslated region (UTR) of target messenger RNAs (mRNAs) and negatively regulate gene expression on the post‐transcriptional level.^10^ Profiling studies have revealed a panel of miRNAs which are deregulated in breast cancer tissue,^11^ and miRNA expression pattern could classify breast tumors by genetic subtypes corresponding to intrinsic molecular subtypes determined by status of estrogen receptor (ER), progesterone receptor (PgR) and human epidermal growth factor receptor 2 (HER2).^12^ Mechanisms such as DNA amplification, deletion, and mutations relating to miRNA loci, epigenetic silencing or inhibition of specific miRNA processing can lead to altered miRNA expression in human cancers.^13^ For instance, let‐7 is often down‐regulated during carcinogenesis. It was found to regulate breast cancer tumor‐initiating cells through targeting HRAS and HMGA2.^14^ MiR‐21, one of the most highly expressed miRNAs in breast cancer, has several targets including tropomyosin 1α and programmed cell death 4 (PDCD4).^15^, ^16^ It also targets PTEN to promote MCF‐7 breast cancer cell growth.^17^, ^18^

The predictive and prognostic value of miRNAs has been investigated in breast cancer.^19^ In a small study (n = 11), Kolacinska et al^20^ found higher expression of miR‐200b‐3p/miR‐190a along with lower expression of miR‐512‐5p in breast cancer tissue before chemotherapy correlated with a better pathologic response to NCT. Wang et al^21^ proposed circulating miR‐125b as a marker predicting chemo‐resistance in breast cancer. In another neoadjuvant study, circulating miR‐375 and miR‐122 exhibited strong correlation with NCT response and metastatic recurrence.^22^

miRNAs could be secreted by tumor cells into the body fluid compartment and referred to as circulating miRNAs,^23^ which are stable, easily accessible, convenient to monitor and thus proposed as potential tumor biomarker.^24^ Research into circulating miRNAs as predictor of response to NCT in breast cancer is limited. In a study enrolling locally advanced and inflammatory breast cancer patients, a two‐gene signature of serum miR‐375 and miR‐122 was found to predict metastatic relapse risk after NCT with a sensitivity of 80% and specificity of 100%.^22^ In addition, miRNAs could be passively released by dead cells into peripheral blood and thus may reflect response to anti‐cancer therapy.^25^ A study by Gezer et al^26^ reported chemo‐induced fluctuation in the levels of multiple serum miRNAs, but the authors failed to further explore the association of such dynamic change with response to NCT.

In this study, we utilized serial plasma samples prospectively collected from breast cancer patients receiving anthracycline‐/taxane‐based NCT to specify the dynamic change in the levels of plasma miRNAs during NCT and explore the association of miRNA dynamics with response to NCT, to identify potential predictive biomarkers of chemo‐sensitivity early in the treatment.

## 2 MATERIALS AND METHODS

### 2.1 Patients

The current work was a correlative study of a prospective randomized clinical trial which aimed to compare the efficacy of different NCT regimens in breast cancer subtypes (ClincalTrials.gov NCT02041338). Patients with newly diagnosed operable or locally advanced breast cancer were recruited at National Cancer Center/Cancer Hospital, Chinese Academy of Medical Sciences (CAMS) from 1/2014 to 11/2015. Exclusion criteria included stage IV disease, bilateral breast cancer, male breast cancer, inflammatory breast cancer, and complication with other malignancies. To minimize the confounding effect of heterogeneity in anti‐cancer treatments, we only enrolled patients from the control arm, which consists of 4‐6 cycles of intravenous ET regimen (Epirubicin 75 g/m^2^ IV on day 1, Paclitaxel 175 mg/m^2^ IV on day 2, every 3 weeks). The study was approved by ethics committee approval from Cancer Hospital CAMS and written informed consent to chemotherapy and blood sample collection was obtained from the participants.

Baseline staging examinations were carried out including bilateral breast MRI or echography, chest X‐ray, abdominal echography or CT scan and bone scintigraphy. Clinical disease response was evaluated for every two cycles of chemotherapy till surgery based on objective measurements obtained through physical and imaging examinations (breast MRI or echography) with the Response Evaluation Criteria in Solid Tumors (RECIST) v1.1.^27^ The same imaging methods were used throughout treatment for a given patient. Pathological response was assessed by two independent pathologists and pathological complete response (pCR) was defined as the absence of invasive carcinoma from both the breast and lymph nodes of the resected specimen.

### 2.2 Study design

This study was performed in a prospective cohort with the aim to explore the relationship between dynamics of plasma miRNAs during NCT and disease response. Peripheral blood samples were collected at baseline, after two cycles of chemotherapy (C2) and before surgery, and the levels of plasma miRNAs were measured. Total pCR rate was low (10/109, 9.2%), which was probably due to exclusion of HER2+ patients receiving trastuzumab with NCT. For HER2+ patients in the clinical trial, trastuzumab was optional in the neoadjuvant setting considering the elevated risk of congestive heart dysfunction/failure with concurrent use of trastuzumab and anthracyclines. Instead, trastuzumab was prescribed alone after surgery for these patients unsuitable for combined use of epirubicin and trastuzumab. To avoid the influence of trastuzumab on miRNA spectrum, patients receiving neoadjuvant anti‐HER2 targeted therapy were excluded from the present analysis. We set objective response as the primary endpoint. Patients with complete/partial remission were defined as chemo‐sensitive and stable/progressive disease as chemo‐insensitive. The study population was classified into three cohorts by differential expression status of HR and HER2, namely HR+/HER2−, HER2+ and triple‐negative breast cancer (TNBC) cohorts, and prospective research was conducted independently within each cohort. The expression status of HR/HER2 was determined by immunohistochemistry (IHC) and fluorescence in situ hybridization (FISH) was conducted to decide the amplification status of *HER2* if IHC revealed HER2++.

**Figure 1:**
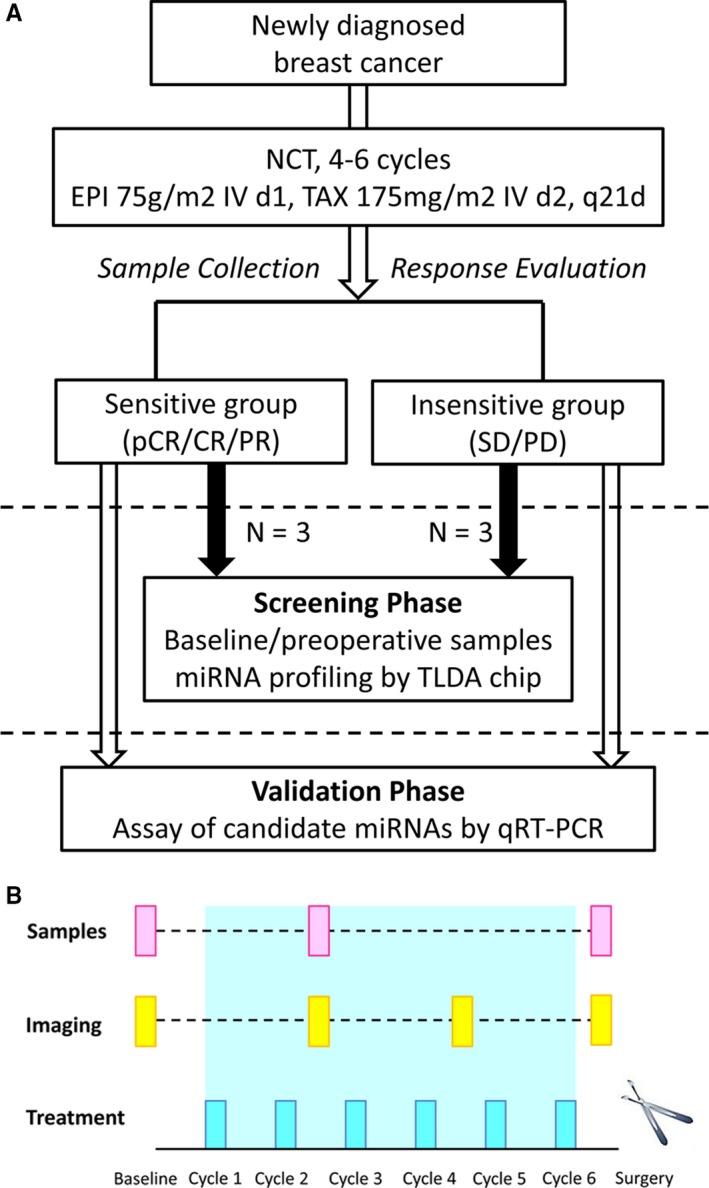
Study design and Schedule for sample collection. A, A two‐phase study was designed. In the screening phase, baseline and preoperative blood samples of selected cases from both groups were screened via microarray for candidate miRNAs whose fluctuations might reflect response. In the validation phase, the fluctuation patterns of candidate miRNAs were confirmed by qRT‐PCR using serially collected blood samples, and the association between dynamics of plasma miRNAs and chemo‐sensitivity was explored. B, For each participant, blood samples were collected at baseline, after two cycles of chemotherapy and before definitive surgery

Overall, a two‐phase study was designed (Figure 1A). First, in the screening stage, three cases were selected from sensitive and insensitive groups of each cohort, respectively. By inter‐group and before/after comparison, baseline and preoperative blood samples of selected cases from both groups were screened via TaqMan low‐density array (TLDA, v3.0, Applied Biosystems, Foster city, CA, USA) chip for candidate miRNAs whose fluctuations might reflect response. In the validation stage, the patterns of candidate miRNAs identified by TLDA chip were confirmed by quantitative real‐time polymerase chain reaction (qRT‐PCR) in the remaining patients using serially collected blood samples, and the association between dynamics of plasma miRNAs and chemo‐sensitivity was interrogated.

### 2.3 Sample preparation and RNA extraction

For each patient, 4‐5 mL of peripheral blood was collected as per predesigned schedule described in Figure 1B. Within one hour of blood drawl, samples were centrifuged at 1200 *g* for 10 minutes at 4°C to separate the plasma supernatant, which was centrifuged for a second time at 12 000 *g* for 10 minutes at 4°C to remove cellular components. Plasma samples were aliquoted and stored at −80°C until analysis.

Total RNAs containing miRNAs were extracted from the plasma using TRIzol LS Reagent (Invitrogen, Carlsbad, CA, USA) following the manufacturer's instructions. Synthetic *C. elegans* miR‐39 (cel‐miR‐39, Applied Biosystems) was added as spike‐in at a final concentration of 80 fmol/μL. RNA samples were quantified using NanoDrop ND‐2000 spectrophotometer (Thermo Scientific, Wilmington, DE, USA).

### 2.4 TaqMan miRNA microarray

For each cohort, we selected three cases from the sensitive and insensitive groups and obtained six couples of self‐paired (baseline and pre‐surgery) plasma samples. A total of 36 TLDA chips were used for the three cohorts. Megaplex RT reactions and pre‐amplification reactions were conducted according to the manufacturer's protocol. Analysis of the qRT‐PCR data was performed using the SDS 2.0.1 software and Data Assist v2.0 software (Applied Biosystems).

### 2.5 qRT‐PCR assays

TaqMan Megaplex RT reactions and pre‐amplification reactions were performed using total RNAs (100 ng) from each sample. Quantitative detections of miRNA, including cel‐miR‐39, were performed using the TaqMan miRNA assay in the StepOne Plus Real‐Time PCR System and fold changes in gene expression were calculated using the 2^−ΔΔ*Ct*^ method.

### 2.6 Statistical analysis

Demographic and clinico‐pathologic characteristics of study population were analyzed using statistical description method. Difference in miRNA levels between groups was evaluated using the Mann‐Whitney unpaired test, and for before/after comparison within one group paired *t*‐test was used. Receiver operating characteristic (ROC) curves were constructed to derive the optimal cut‐off values for candidate miRNAs. Multivariate logistic model was built to assess the independent predictors of clinical response, including age, status of menopause, clinical tumor stage, clinical node stage, clinical TNM stage, grade and miRNA expression levels. Disease‐free survival (DFS) was defined as the interval between initiation of NCT and the date of disease relapse or death from any cause. Overall survival (OS) was calculated from the date of treatment initiation to the date of death. Cases without relapse or death events were censored at the date of last follow‐up. Survival curves were estimated using the Kaplan‐Meier method and unadjusted comparison of these estimates was made using log‐rank test. All statistical analyses were performed using SPSS software v19.0. All *P* values were bilateral, with *P* < 0.05 being statistically significant.

## 3 RESULTS

### 3.1 Patient characteristics and response to NCT

In all, 109 patients were included in this study (Table 1). The median age was 48 years old (range 23‐60 years). 71 women (65.1%) were premenopausal. The majority was diagnosed with locally advanced disease. Total pCR rate was 9.2%. In terms of objective response, 31.2% of the patients were chemo‐insensitive (stable disease (SD)/progressive disease (PD)). In further subtype‐based analysis, disparity in sensitivity to chemotherapy was observed in different cohorts. The rate of insensitive cases was 31.6% (18/57), 23.3% (7/30) and 40.9% (9/22) for the HR+/HER2−, HER2+ and TNBC cohort, respectively. 27.5% (30/109) of the study population received breast‐conserving surgery. Given that there might be subtype‐dependent variation in miRNA profiling results, prospective study was carried out separately in each cohort.

**Table 1:** Baseline characteristics and response to neoadjuvant chemotherapy

Characteristics	Number (%)
Age (y)	
≤50	64 (58.7)
>50	45 (41.3)
Menopause	
Premenopausal	71 (65.1)
Postmenopausal	38 (34.9)
Histology	
Invasive ductal carcinoma(IDC)	105 (96.3)
Others	4 (3.7)
Grade	
I/II	90 (82.6)
III	19 (17.4)
HR	
Positive	76 (69.7)
Negative	33 (30.3)
HER2	
Positive	30 (27.5)
Negative	79 (72.5)
Subtypes	
HR+/HER2−	57 (52.3)
HER2+	30 (27.5)
TNBC	22 (20.2)
Clinical tumor staging	
cT1/2	60 (55.0)
cT3/4	49 (45.0)
Clinical node staging	
cN0	10 (9.2)
cN1	28 (25.7)
cN2	45 (41.3)
cN3	26 (23.8)
Clinical TNM staging	
II	24 (22.0)
III	85 (78.0)
Pathological response	
pCR	10 (9.2)
Non‐pCR	99 (90.8)
Objective response	
CR/PR	75 (68.8)
SD/PD	34 (31.2)

### 3.2 Screening stage

Within each subtype, three sensitive and insensitive cases were selected, whose baseline and preoperative plasma samples were sent for TaqMan miRNA array (n = 12). Shown in Table 2 were clinico‐pathologic characteristics of chosen subjects, which were equally distributed among sensitive and insensitive cases.

**Table 2:** Characteristics of subjects selected for TaqMan microRNA array

No.	Age	Histology	Grade	Subtype	cTNM	Response	Sensitivity
1	46	IDC	II	HR+/HER2−	IIIA	PR	Sensitive
2	56	IDC	II	HR+/HER2−	IIIA	PR	Sensitive
3	56	IDC	II	HR+/HER2−	IIIA	pCR	Sensitive
4	40	IDC	II	HR+/HER2−	IIIA	SD	Insensitive
5	53	IDC	II	HR+/HER2−	IIA	SD	Insensitive
6	49	IDC	II	HR+/HER2−	IIIC	SD	Insensitive
7	42	IDC	III	HER2+	IIIC	PR	Sensitive
8	51	IDC	III	HER2+	IIIA	pCR	Sensitive
9	55	IDC	II	HER2+	IIB	pCR	Sensitive
10	46	IDC	II	HER2+	IIB	SD	Insensitive
11	37	IDC	II	HER2+	IIIC	SD	Insensitive
12	39	IDC	II	HER2+	IIIB	PD	Insensitive
13	50	IDC	II	TNBC	IIB	PR	Sensitive
14	58	IDC	II	TNBC	IIIC	PR	Sensitive
15	55	IDC	III	TNBC	IIIA	pCR	Sensitive
16	42	IDC	II	TNBC	IIIA	SD	Insensitive
17	30	IDC	III	TNBC	IIB	SD	Insensitive
18	54	IDC	III	TNBC	IIIC	PD	Insensitive

**Figure 2:**
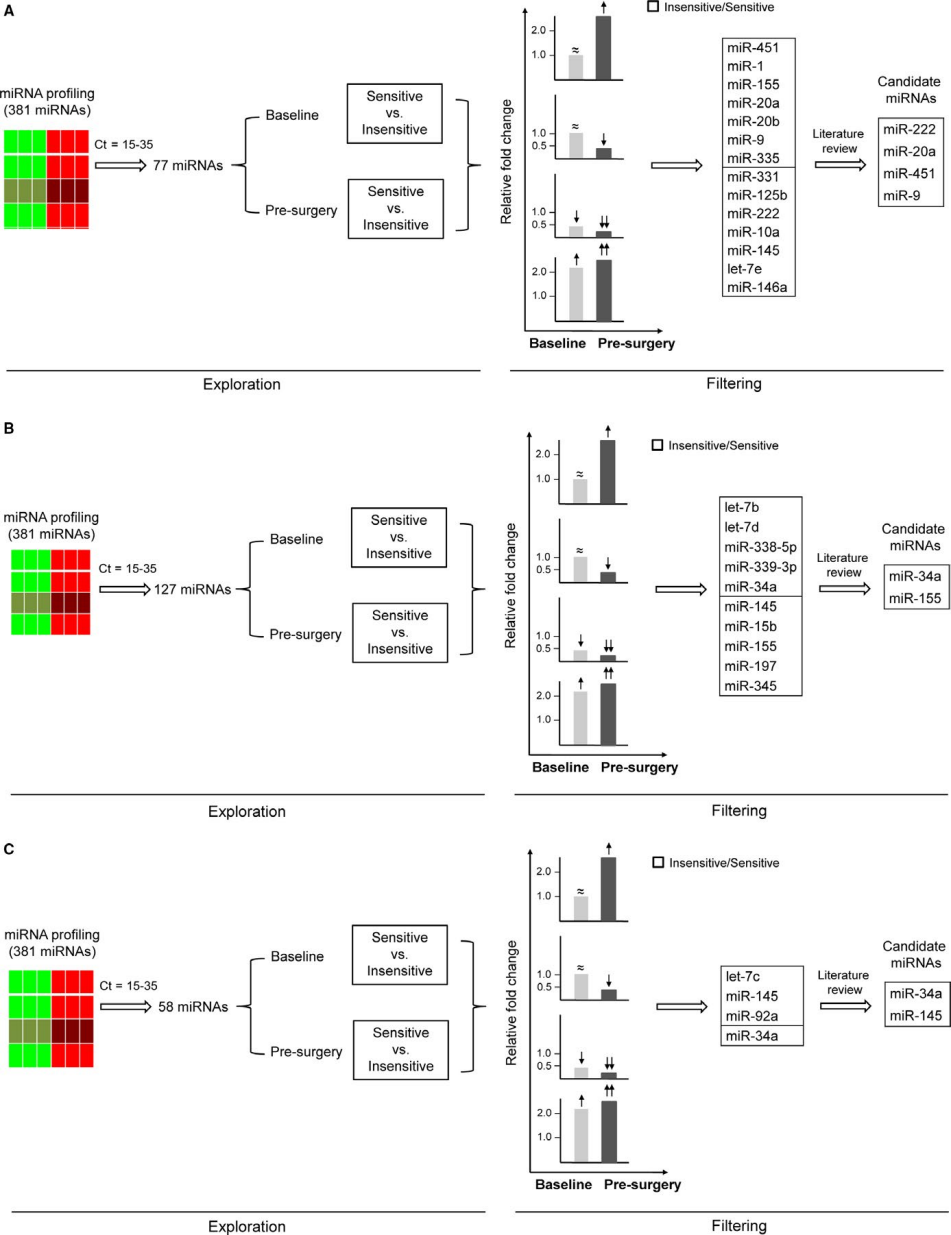
Workflow diagram for identification of candidate miRNAs in HR+/HER2‐ (A), HER2+ (B) and TNBC (C) cohorts. Within each subtype, three sensitive and insensitive cases were selected, whose baseline and preoperative plasma samples were sent for TaqMan miRNA array (n = 12). Among all human miRNAs screened via TLDA chips, only those miRNAs that had *C*_*t*_ values of 15‐35 in all the twelve samples were further analyzed. Two patterns of fluctuation in plasma miRNAs might correlate with chemo‐sensitivity. First, no apparent inter‐group difference was observed pretreatment (indicated by ≈), yet chemo‐induced contrary trends of change (indicated by ↑ or ↓) was exhibited in sensitive and insensitive groups. Secondly, miRNAs were differentially expressed (>2‐fold (indicated by ↑) or <0.5‐fold (indicated by ↓) altered expression) between sensitive and insensitive groups at baseline, and intergroup discrepancy further expanded (indicated by ↑↑ or ↓↓) in the presurgery samples. Through literature review candidate miRNAs were determined and subjected to individual qRT‐PCR confirmation. The bars represent the relative miRNA expression of insensitive versus sensitive group at baseline (gray) and presurgery (black)

**Figure 3:**
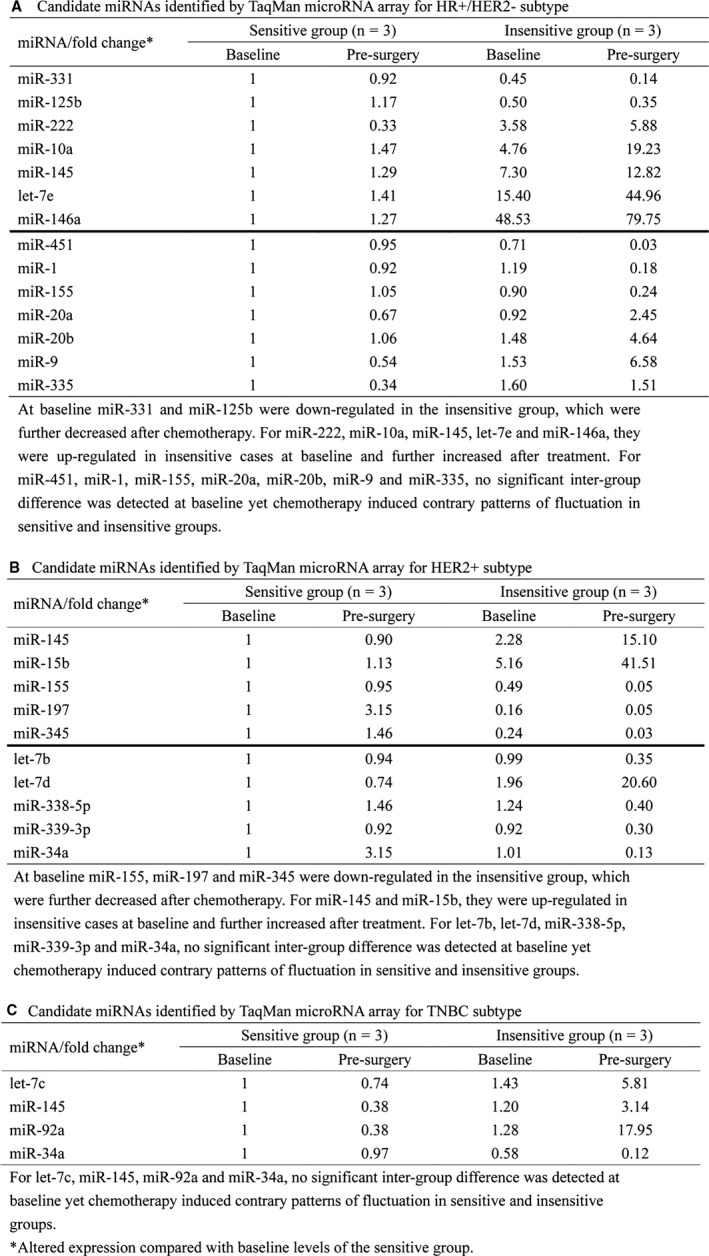
Candidate miRNAs identified by TaqMan microRNA array for HR+/HER2‐ subtype (A), HER2+ (B) and TNBC (C) cohorts

For HR+/HER2− cohort, among all human miRNAs screened via TLDA chips only those miRNAs that had Ct values of 15‐35 in all the twelve samples (n = 77) were further analyzed. Cel‐miR‐39 was used as a control for normalization. With an effort to identify differentially expressed miRNAs in baseline samples between sensitive and insensitive cases and between self‐paired baseline and presurgery samples, we disclosed two patterns of fluctuation in plasma miRNAs that might correlate with chemo‐sensitivity (Figure 2A). First, 34 miRNAs were differentially expressed (>2‐fold or <0.5‐fold altered expression) between sensitive and insensitive groups at baseline, and for 7 of them inter‐group discrepancy further expanded in the presurgery samples (including miR‐331, miR‐125b, miR‐222, miR‐10a, miR‐145, let‐7e, and miR‐146a). Secondly, for 33 miRNAs no apparent inter‐group difference was observed pretreatment, yet 7 of them exhibited chemo‐induced contrary trends of change in sensitive and insensitive groups (including miR‐451, miR‐1, miR‐155, miR‐20a, miR‐20b, miR‐9, and miR‐335). Levels of above 14 miRNAs in baseline and presurgery plasma samples were shown in Figure 3A. Through literature review we narrowed down the list of miRNAs following the criteria that (a) cells of origin have been proposed; (b) reported in breast cancer‐related research; (c) targets/implicated pathways have been described. Finally, miR‐222, miR‐451, miR‐20a, and miR‐9 were subjected to individual qRT‐PCR confirmation.

Candidate miRNAs were determined according to the workflow described above for the other subtypes. In HER2+ cohort (Figure 2B and 3B), let‐7b, let‐7d, miR‐338‐5p, miR‐339‐3p, and miR‐34a displayed diverging patterns of change in two groups after chemotherapy, and for miR‐155, miR‐145, miR‐15b, miR‐197, and miR‐345, inter‐group difference which was already present further expanded after treatment. MiR‐34a and miR‐155 were chosen as candidates. In TNBC cohort (Figure 2C and 3C), the dynamics of miR‐34a and miR‐145 were validated in subsequent phase of study.

### 3.3 Validation stage

**Figure 4:**
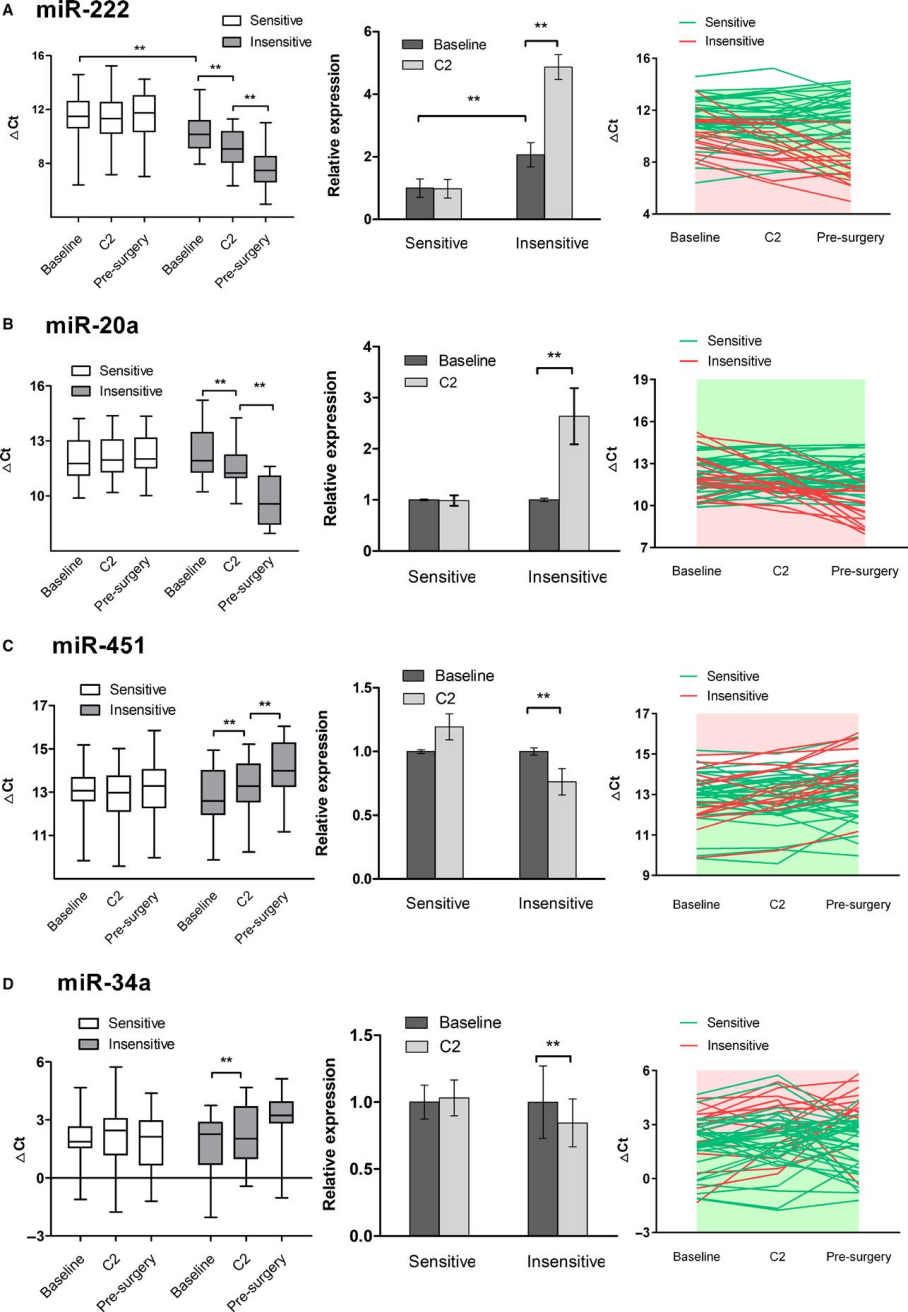
Dynamic change of plasma miRNAs during NCT in sensitive (n = 36) and insensitive (n = 15) groups in HR+/HER2‐ cohort. Expression of plasma miR‐222 (A), miR‐20a (B), miR‐451 (C) and miR‐34a (D) was determined by qRT‐PCR in serially collected blood samples. Delta Ct was calculated using cel‐miR‐39 as an exogeneous control. Relative expression was measured by fold change. The data are expressed as mean ± SEM. **P value < 0.05

In HR+/HER2− cohort (n = 51), fluctuation patterns for three of four candidate miRNAs were consistent with microarray findings. As shown in Figure 4A, at baseline plasma miR‐222 was significantly up‐regulated in the insensitive group (2.065‐fold, *P* = 0.047), which was further elevated after C2 (4.870‐fold, *P* < 0.001) and completion of chemotherapy (*P* = 0.004, compared with C2). Such pattern was not detected in the sensitive group (C2 vs. baseline, 0.977‐fold, *P* = 0.826). As for miR‐20a (Figure 4B), no significant inter‐group difference was detected at baseline (*P* = 0.218), but in the insensitive patients we observed treatment‐induced up‐regulation (C2 vs. baseline, 2.637‐fold, *P* = 0.008) which was absent from the sensitive ones (C2 vs. baseline, 0.986‐fold, *P* = 0.882). As shown in Figure 4C, dynamics of plasma miR‐451 were contrary to that of miR‐20a, with therapy‐related down‐regulation of target miRNA being identified among the insensitive patients (C2 vs. baseline, 0.762‐fold, *P* = 0.014). Regarding miR‐9, we failed to observe apparent fluctuation after C2 within both groups, but in the sensitive group further decrease in plasma miR‐9 level was identified before surgery (Figure S1A).

**Figure 5:**
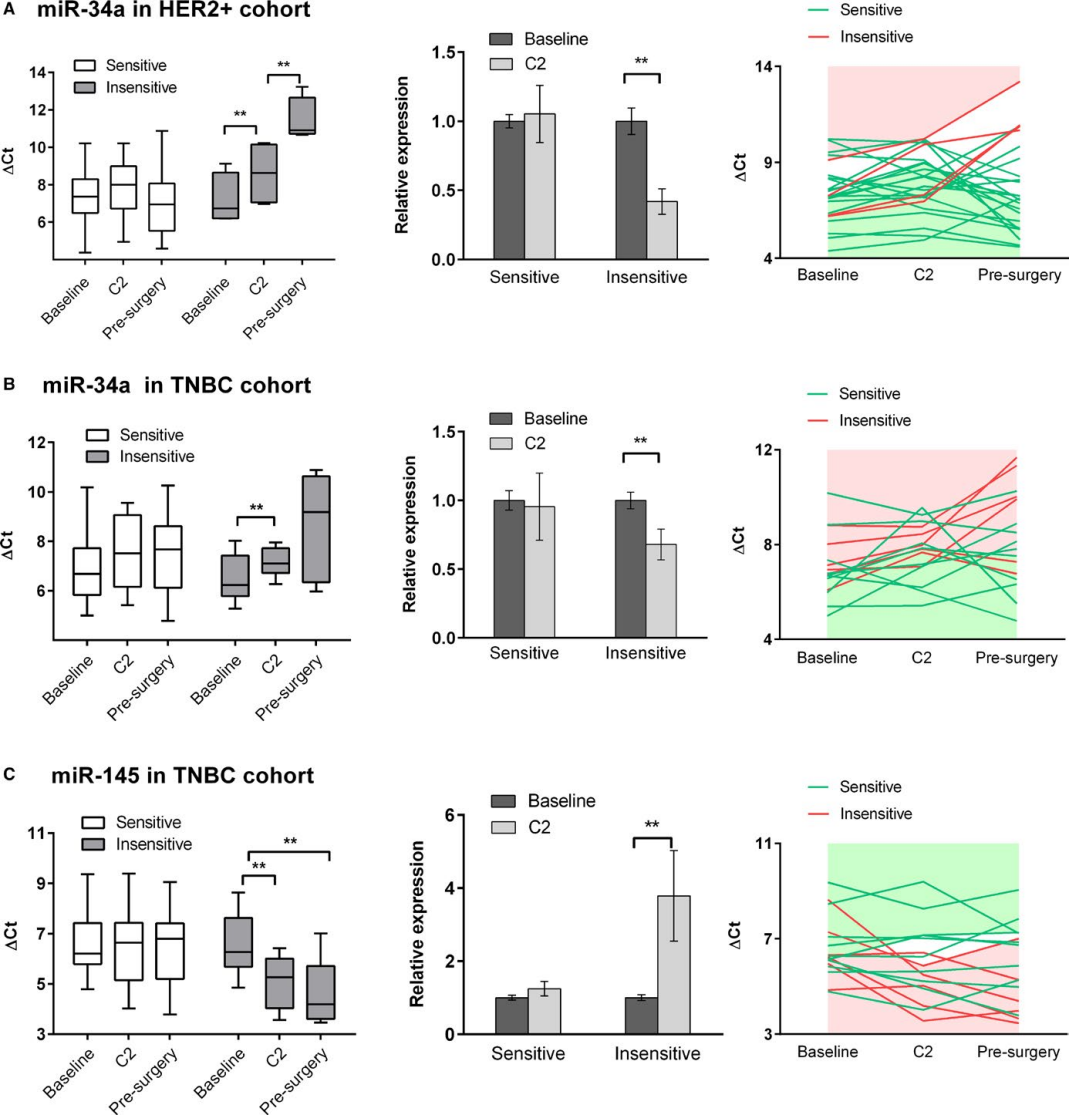
Dynamic change of plasma miRNAs during NCT. Expression of plasma miRNAs was determined by qRT‐PCR in serially collected blood samples. Delta Ct was calculated using cel‐miR‐39 as an exogeneous control. Relative expression was measured by fold change. The data are expressed as mean ± SEM. **P value < 0.05. A, Dynamics of plasma miR‐34a in sensitive (n = 20) and insensitive (n = 4) groups in HER2+ cohort. B, Dynamics of plasma miR‐34a in sensitive (n = 10) and insensitive (n = 6) groups in TNBC cohort. C, Dynamics of plasma miR‐145 in sensitive (n = 10) and insensitive (n = 6) groups in TNBC cohort

In the HER2+ cohort (n = 24), four patients were defined as chemo‐insensitive. For plasma miR‐34a, findings from qRT‐PCR of serial samples (Figure 5A) were consistent with those of array. MiR‐34a levels were similar in both groups at baseline (*P* = 0.757). In the insensitive group, markedly decreased expression of plasma miR‐34a was detected after two cycles of chemotherapy (C2 vs. baseline, 0.419‐fold, *P* = 0.027). Along with cycles adding, the expression of miR‐34a was further suppressed (presurgery vs. C2, *P* = 0.030). For chemo‐sensitive patients, the levels of miR‐34a were relatively stable throughout the course of treatment (*P* = 0.782). Yet for miR‐155 little, if any, change was detected throughout NCT in both groups (Figure S1B).

In the TNBC cohort, 6 of 16 patients were insensitive to chemotherapy. Dynamics of two candidate miRNAs coincided with those revealed by TLDA chips. The dynamic change of miR‐34a was also found to correlate with chemo‐sensitivity of patients with TNBC (Figure 5B). Treatment‐induced noteworthy down‐regulation of miR‐34a in the insensitive group (C2 vs. baseline, 0.680‐fold, *P* = 0.006), while in the sensitive patients the levels of miR‐34a remained relatively stable despite administration of chemotherapy (C2 vs. baseline, 0.955‐fold, *P* = 0.836). With respect to plasma miR‐145, results produced by array were also validated in subsequent individual assay. As displayed in Figure 5C, up‐regulation of miR‐145 after treatment was observed in the insensitive group (C2 vs. baseline, 3.789‐fold, *P* = 0.032) rather than the sensitive group (C2 vs. baseline, 1.247‐fold, *P* = 0.272).

Similar patterns of fluctuation in plasma miR‐34a were found in the HER2+ and TNBC cohorts, implying that the association between dynamics of miR‐34a and chemo‐sensitivity might be subtype‐independent. We further explored that in the HR+/HER2− cohort and derived similar findings (Figure 4D). After two cycles of therapy decreased expression of plasma miR‐34a was observed in the insensitive patients (C2 vs. baseline, 0.845‐fold, *P* = 0.049), but in the sensitive group no significant change in the level of miR‐34a was detected (C2 vs. baseline, 1.032‐fold, *P* = 0.855).

### 3.4 Predictive value of miRNA dynamics

**Figure 6:**
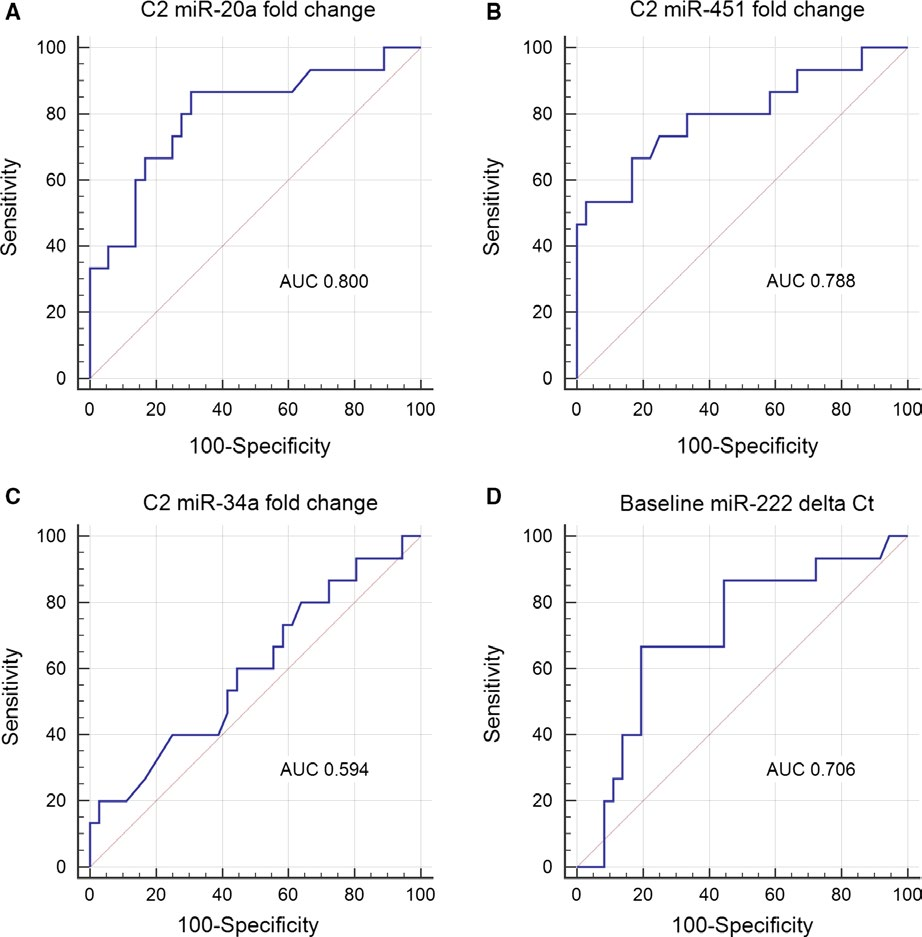
ROC curves for baseline/C2 expression of plasma miRNAs in HR+/HER2‐ cohort

To evaluate the value of the miRNAs in predicting response to NCT in breast cancer, ROC curves were utilized to derive the optimal cut‐off value for each miRNA. We set baseline delta *C*_t_ value as the variable for the set of miRNAs with differential expression before treatment, and C2 fold change (compared with baseline) for those with chemotherapy‐induced altered expression. For miR‐222, at baseline, we detected a response‐associated difference in its plasma level, which is represented directly by delta *C*_t_. For the other miRNAs, however, the dynamic change after two cycles was found to correlate with response. This fold change, or relative expression, was calculated by 2^−ΔΔ*C*t^ method. These were potential factors predictive of clinical response and thus incorporated into the logistic regression model along with other clinicopathological variables. In the HR+/HER2− cohort four miRNAs were analyzed (Figure 6). The area under the curve (AUC) of individual miRNA ranged from 0.594 to 0.800. Plasma miR‐222 could be grouped into low or high expression based on pretreatment delta *C*_t_ value (cut‐off value 11.3). Modified expression of miR‐20a, miR‐451 and miR‐34a was measured by C2 fold change (cut‐off values were 1.25, 0.78 and 0.95 respectively) and defined as up‐regulated or down‐regulated. By multivariate logistic regression model, baseline miR‐222 (OR = 6.422, *P* = 0.049), C2 miR‐20a (OR = 0.144, *P* = 0.021) and C2 miR‐451 (OR = 8.213, *P* = 0.012) were demonstrated to be indicative of objective response to NCT in HR+/HER2− breast cancer.

The predictive role of circulating miRNA dynamics was also assessed in the HER2+ and TNBC cohorts. Probably due to compromised sample size and small number of events, logistic regression analysis revealed that plasma miR‐34a and miR‐145 were not independent predictors of clinical response.

We further evaluated the prognostic value of selected miRNAs. By the last follow‐up date (March 1st, 2018), 23 (21.1%) disease relapse and 10 (9.2%) death events were reported in the whole population. For C2 miR‐34a, although its down‐regulation was related with insensitivity to NCT across all three subtypes, we failed to observe association of this biomarker with survival (Figure S3A‐B). In the HR+/HER2− cohort, baseline miR‐222, C2 miR‐20a and C2 miR‐451 were unable to discriminate the prognosis of the patients, in terms of either DFS or OS (Figure S3C‐H).

## 4 DISCUSSION

In the present study, by serial circulating miRNA assay we described that expression of plasma miRNAs could be modified by NCT in breast cancer. Consistent with published reports,^28^, ^29^ our study showed subtype‐related disparity in sensitivity to cytotoxic agents, with HR+/HER2− breast cancer and TNBC being less responsive to anthracycline/taxane‐containing therapy, which provided rationale for identification of biomarkers predictive of response. Our analysis revealed three plasma miRNAs as predictors of chemo‐sensitivity in HR+/HER2− cohort. High level of plasma miR‐222 at baseline was associated with poor response to NCT, and chemotherapy led to further increase of miR‐222 in insensitive patients, implying that miR‐222 might be involved in the mechanism underpinning resistance to anthracyclines/taxanes. Baseline levels of circulating miR‐20a and miR‐451 did not correlate to chemo‐sensitivity but their dynamics (C2 fold change) predicted the ultimate clinical response, supporting these two miRNAs as early markers of response to NCT.

As an oncogenic factor, miR‐222 was found to inhibit the expression of tricho‐rhino‐phalangeal syndrome type 1 protein (TRPS1) and promote the process of epithelial‐to‐mesenchymal transition (EMT), leading to acquisition of chemo‐resistance and formation of aggressive phenotype of breast cancer.^30^ Another study showed that miR‐222 was highly enriched in the exosomes secreted by chemo‐resistant breast cancer cells (MCF‐7/Adr and MCF‐7/Doc). After being cocultured with exosomes from resistant cells, chemo‐sensitive MCF‐7 cells exhibited decreased sensitivity to cytotoxic agents, along with increased intracellular miR‐222 and reduced PTEN level.^31^ Our results, together with previous lab findings, might provide hints to the role of miR‐222 in chemo‐resistance of breast cancer. Lesions from insensitive patients might contain miR‐222‐overexpressed chemo‐resistant clones (evidenced by higher level of plasma miR‐222 before treatment in the insensitive cases), which could secrete miR‐222‐enriched exosomes into the bloodstream under the pressure of cytotoxic agents (evidenced by chemo‐induced up‐regulation of plasma miR‐222). Exosomes encapsulating miR‐222 might be taken in by sensitive clones, transmitting chemo‐resistance to sensitive cells and resulting in poor response. This hypothesis, however, needs to be confirmed in future research.

Our findings have proposed C2 miR‐20a up‐regulation and C2 miR‐451 down‐regulation as early markers of chemo‐sensitivity in HR+/HER2− breast cancer. Previous data indicated these two miRNAs might be players mediating resistance in breast cancer. MiR‐451 regulates the expression of multidrug resistance protein 1 (MRP‐1) which facilitates insensitivity to anthracyclines, and MCF‐7/Adr cells enforced with overexpression of miR‐451 recovered cellular chemo‐sensitivity.^32^ MiR‐451 can promote mechanistic target of rapamycin (mTOR) activity and mediate cell energy‐consuming models,^33^ in vitro studies proposed that it was abundantly released by breast cancer cells and enriched extracellularly.^23^ A recent study showed that miR‐451 is abundantly present in most tissues and is from only red blood cells (RBC).^34^ The finding of C2 miR‐451 down‐regulation may be due to the low number of RBC in tumors or the lower number of miR‐451 that was released (passive or active) from the similar number of RBC in tumors, which might warrant further study in the future. MiR‐20a, which belongs to miR‐17‐92 cluster, was regulated by c‐Myc and involved in tumorigenesis in breast cancer.^35^ MiR‐20a could inhibit the expression of tumor‐suppressor ZBTB4 protein, which was related to prognosis of breast cancer.^36^ We observed, for the first time to our knowledge, the relationship between miR‐20a and response to chemotherapy in breast cancer, and the underlying molecular mechanism warrants further studies.

Another major finding of our study was the association between C2 miR‐34a down‐regulation and response to NCT across all subtypes of breast cancer, which suggested that plasma miR‐34a was a potential subtype‐independent marker of clinical response to NCT. Compared with sensitive group, insensitive patients presented similar levels of plasma miR‐34a at baseline, but after treatment miR‐34a was down‐regulated, indicating its tumor‐suppressive role. Considering that the relationship between C2 miR‐34a change and chemo‐sensitivity seemed subtype‐independent, we sought to evaluate its predictive role in the whole population. The AUC was 0.699 (Figure S2), suggesting mild to moderate indicative value for this marker. In a study which included 25 breast cancer patients receiving NCT with anthracyclines and achieving pathological partial response (pPR) or pCR, the authors observed up‐regulation of both plasma and tumor miR‐34a after chemotherapy. For one patient who was not responding to NCT (ypT2N2Mx), miR‐34a level significantly decreased after treatment.^37^ Earlier research proved that the transcription factor p53 directly regulated the expression of miR‐34a by binding to its promoter,^38^, ^39^ and miR‐34a could further facilitate cellular apoptosis by targeting Bcl‐2 and SIRT1.^40^ In addition, in vitro studies showed down‐regulated cellular miR‐34a in breast cancer cells with acquired resistance to anthracyclines^41^ and that forced overexpression of miR‐34a could reverse drug resistance.^42^ These evidences aided in better interpretation of our finding. Cytotoxic agents could cause apparent DNA damage in chemo‐sensitive cancer cells and might up‐regulate cellular miR‐34a via p53‐(in)dependent pathway. Yet with treatment cycles adding and tumor being debulked, the levels of circulating miR‐34a might remain stable in the sensitive patients. On the other hand, for insensitive patients miR‐34a in the tumor tissue might be down‐regulated through unknown mechanism (eg, DNA overmethylation^43^), leading to reduced levels of plasma miR‐34a after chemotherapy. This preliminary speculation needs verification in fundamental research. Above all, our results indicated that the dynamic change in circulating miR‐34a was a potential marker of chemo‐sensitivity.

We also compared our findings to studies evaluating miRNAs as predictive biomarkers in other types of cancers. In HER2‐positive gastric cancer, Sui et al^44^ has demonstrated that increased miR‐125b level in tumor tissue was significantly associated with trastuzumab resistance, advanced malignant progression, as well as poor prognosis. In vitro studies revealed that miR‐21/PTEN and miR‐223/FBXW7 pathways might be implicated in sensitivity of gastric cancer to trastuzumab.^45^, ^46^ For other HR‐positive cancers like ovarian cancer, data regarding miRNA change during chemotherapy or after surgery is lacking. Several studies using clinical material revealed association of miRNA levels with chemotherapy response,^47^, ^48^, ^49^, ^50^ although there is poor reproducibility between studies, which may be partly explained by the different microarray platforms adopted and different categorization of chemotherapy response.

The present research features several improvements over previous studies. First and foremost, our study represented an attempt to specify the relationship between the dynamics of circulating miRNAs and response to NCT in breast cancer. Via serial assay of circulating miRNAs during NCT, we determined the major patterns of fluctuation in plasma miRNAs and the predictive value of plasma miRNA dynamics, proposing a novel indicator of response that had the potential to guide personalized delivery of NCT. Our results exemplified the clinical utility of serial plasma miRNA assay and provided further insights into the molecular biology underlying chemo‐resistance. Secondly, the present study was a translational project based on a prospective clinical trial. All patients were given uniform treatment, and separate exploration and blinded analysis were carried out in each subtype of breast cancer, minimizing the confounding effect of heterogeneity in treatment and disease biology. Besides, we selected cel‐miR‐39 spike‐in as the exogeneous control to avoid the influence of normal tissue or comorbidities and generate stably reproducible results. The major limitations of our study should also be highlighted. The power of analysis was compromised because of small capacity and number of events, especially in the HER2+ and TNBC cohorts. Moreover, it should be admitted that ET regimen is not among the standard chemotherapy regimens of breast cancer, especially in the absence of trastuzumab prescription in HER2‐positive patients. Importantly, the costs and variable accuracy of serial miRNA assay preclude its clinical implication, which might be overcome with technology improvement. Given that dynamics of plasma miR‐34a might serve as a potential marker of chemo‐sensitivity, its clinical relevance should be further validated in larger series of patients with breast cancer. Our results warrant further validation study in larger breast cancer patient cohort.

## CONFLICT OF INTEREST

The authors disclose no potential conflict of interest.
