# Supplementary figures and images for: Dynamics of circulating microRNAs as a novel indicator of clinical response to neoadjuvant chemotherapy in breast cancer
Source: Cancer Med. 2018 Aug 11;7(9):4420–33. doi: 10.1002/cam4.1723 (PMC6144164; doi:10.1002/cam4.1723)

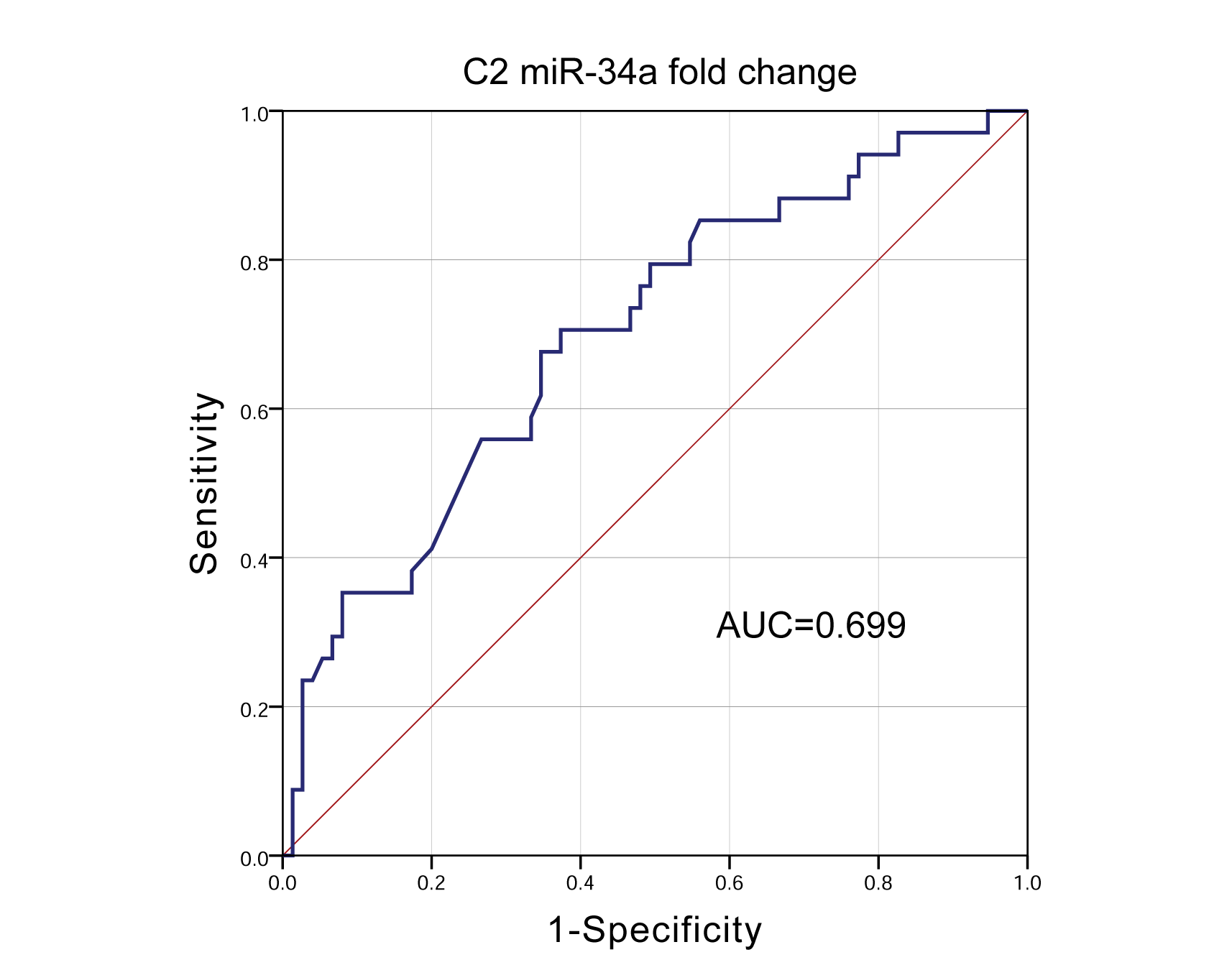

Supplement: Supplementary file 2 [file CAM4-7-4420-s002.tif]

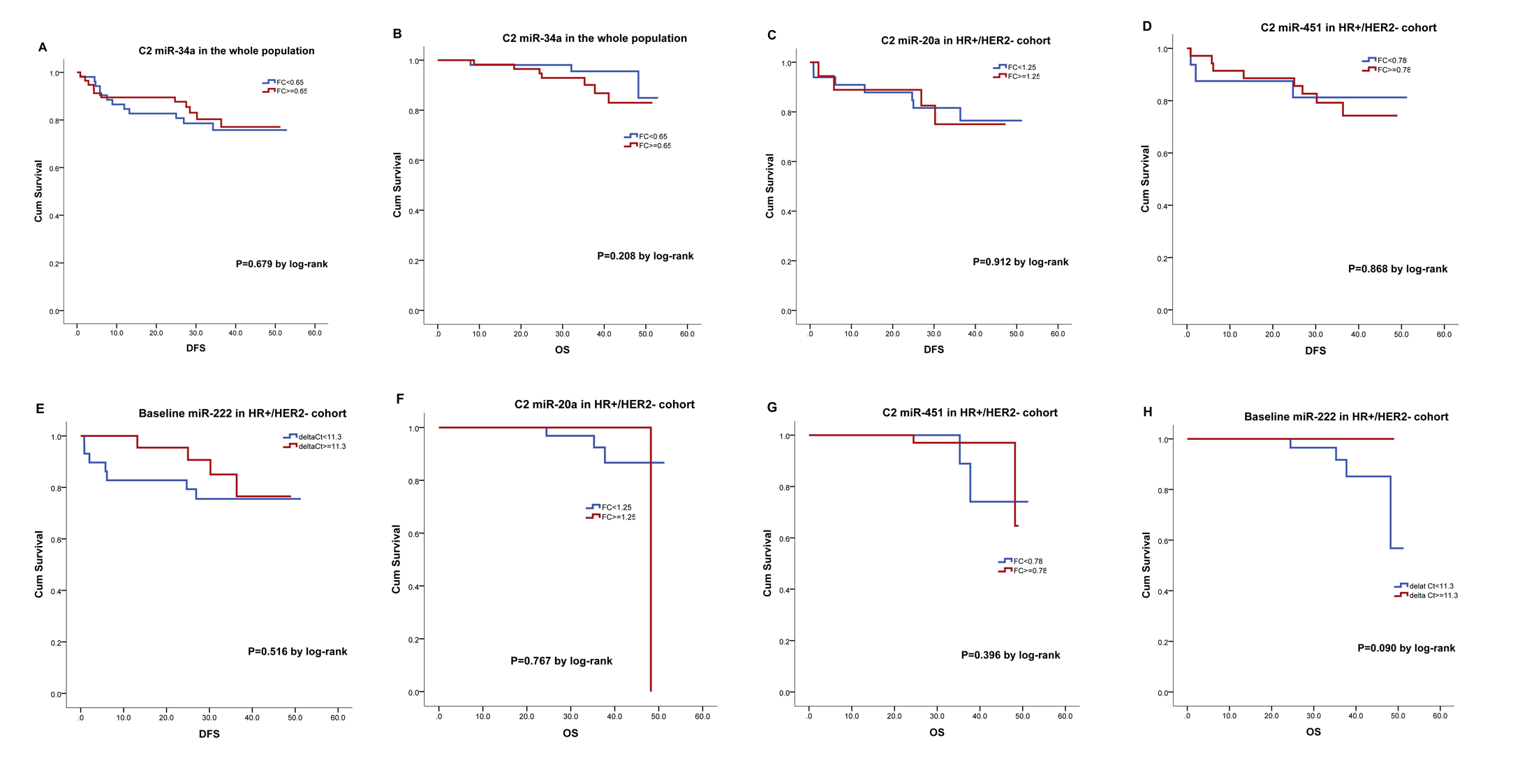

Supplement: Supplementary file 3 [file CAM4-7-4420-s003.tif]
