# Supplementary material for: Dynamics of circulating microRNAs as a novel indicator of clinical response to neoadjuvant chemotherapy in breast cancer
Source: Cancer Med. 2018 Aug 11;7(9):4420–33. doi: 10.1002/cam4.1723 (PMC6144164; doi:10.1002/cam4.1723)
Supplement: Supplementary file 4 [file CAM4-7-4420-s004.docx]

**Supplementary Table 1 Logistic regression model in HR+/HER2- breast cancer (N=51)**

| **variables** | **Univariate logistic regression** | | | **Multivariate logistic regression** | | |
| --- | --- | --- | --- | --- | --- | --- |
|  | **OR** | **95% CI** | **P value** | **OR** | **95% CI** | **P value** |
| age | 0.50 | 0.15-1.73 | 0.276 | -- | -- | -- |
| menopause | 0.77 | 0.21-2.82 | 0.692 | -- | -- | -- |
| BMI | 1.60 | 0.45-5.63 | 0.464 | -- | -- | -- |
| histology | 7.16 | 0.72-71.55 | 0.094 | -- | -- | -- |
| grade | 2.26 | 0.24-21.17 | 0.476 | -- | -- | -- |
| Ki-67 | 0.99 | 0.97-1.02 | 0.565 | -- | -- | -- |
| cT | 1.23 | 0.37-4.12 | 0.743 | -- | -- | -- |
| cN | 0.79 | 0.20-3.15 | 0.733 | -- | -- | -- |
| cTNM stage | 0.25 | 0.03-2.20 | 0.212 | -- | -- | -- |
| miR-222 | 8.13 | 1.60-41.36 | **0.012** | 6.42 | 1.01-41.03 | **0.049** |
| C2 miR-20a | 0.14 | 0.04-0.54 | **0.004** | 0.14 | 0.03-0.74 | **0.021** |
| C2 miR-451 | 10.00 | 2.50-39.98 | **0.001** | 8.21 | 1.59-42.46 | **0.012** |
| C2 miR-34a | 2.26 | 0.54-9.51 | 0.266 | -- | -- | -- |

**Supplementary Table 2 Logistic regression model in HER2+ breast cancer (N=24)**

| **variables** | **Univariate logistic regression** | | | **Multivariate logistic regression** | | |
| --- | --- | --- | --- | --- | --- | --- |
|  | **OR** | **95% CI** | **P value** | **OR** | **95% CI** | **P value** |
| age | 2.29 | 0.37-14.32 | 0.375 | -- | -- | -- |
| menopause | 1.61 | 0.26-10.13 | 0.614 | -- | -- | -- |
| BMI | 1.22 | 0.22-6.73 | 0.818 | -- | -- | -- |
| grade | 2.04 | 0.26-22.19 | 0.986 | -- | -- | -- |
| Ki-67 | 0.93 | 0.87-0.99 | **0.029** | 0.94 | 0.87-1.02 | 0.121 |
| cT | 0.58 | 0.10-3.33 | 0.544 | -- | -- | -- |
| cN | 0.37 | 0.06-2.23 | 0.278 | -- | -- | -- |
| cTNM stage | 1.13 | 0.17-7.47 | 0.896 | -- | -- | -- |
| C2 miR-34a | 0.00 | 0.00-0.54 | **0.029** | 0.01 | 0.00-1.35 | 0.064 |

**Supplementary Table 3 Logistic regression model in triple-negative breast cancer (N=16)**

| **variables** | **Univariate logistic regression** | | | **Multivariate logistic regression** | | |
| --- | --- | --- | --- | --- | --- | --- |
|  | **OR** | **95% CI** | **P value** | **OR** | **95% CI** | **P value** |
| age | 1.00 | 0.13-7.57 | 1.000 | -- | -- | -- |
| menopause | 0.43 | 0.05-3.48 | 0.428 | -- | -- | -- |
| BMI | 5.00 | 0.42-59.66 | 0.203 | -- | -- | -- |
| grade | 7.50 | 0.62-90.65 | 0.113 | -- | -- | -- |
| Ki-67 | 0.96 | 0.91-1.01 | 0.090 | -- | -- | -- |
| cT | 1.33 | 0.16-11.08 | 0.790 | -- | -- | -- |
| cN | 0.22 | 0.02-3.22 | 0.270 | -- | -- | -- |
| cTNM stage | 9.00 | 0.66-122.79 | 0.099 | -- | -- | -- |
| C2 miR-145 | 2.88 | 0.84-9.88 | 0.093 | -- | -- | -- |
| C2 miR-34a | 0.43 | 0.06-3.14 | 0.405 | -- | -- | -- |

**Figure S1 Dynamic change of plasma miR-9 and miR-155 during NCT in sensitive and insensitive groups.** Expression of plasma miR-9 (A) and miR-155 (B) was determined by qRT-PCR in serially collected blood samples. Delta Ct was calculated using cel-miR-39 as an exogeneous control. Relative expression was measured by fold change. The data are expressed as mean±SEM. ** indicates P value<0.05

**Figure S2** **ROC curve for C2 expression of plasma miR-34a in the whole population.**

**Figure S3 Kaplan-Meier survival curves of the patients stratified by selected miRNA change.** Unadjusted comparison was made using log-rank test.
